# ADAR1调节ERK/c-FOS/MMP-9通路驱动非小细胞肺癌细胞的增殖和迁移

**DOI:** 10.3779/j.issn.1009-3419.2025.106.26

**Published:** 2025-09-20

**Authors:** Li ZHANG, Xue PAN, Wenqing YAN, Shuilian ZHANG, Chiyu MA, Chenpeng LI, Kexin ZHU, Nijia LI, Zizhong YOU, Xueying ZHONG, Zhi XIE, Zhiyi LV, Weibang GUO, Yu CHEN, Danxia LU, Xuchao ZHANG

**Affiliations:** ^1^510080 广州，南方医科大学附属广东省人民医院（广东省医学科学院）; ^1^Guangdong Provincial People's Hospital (Guangdong Academy of Medical Sciences), Southern Medical University, Guangzhou 510080, China; ^2^510515 广州，南方医科大学附属第二临床医学院; ^2^The Second School of Clinical Medicine, Southern Medical University, Guangzhou 510515, China

**Keywords:** 肺肿瘤, 非小细胞肺癌, 肿瘤转移, ADAR1, MMP-9, Lung neoplasms, Non-small cell lung cancer, Tumor metastasis, ADAR1, MMP-9

## Abstract

**背景与目的:**

双链RNA特异性腺苷脱氨酶1（double-stranded RNA-specific adenosine deaminase 1, ADAR1）能结合和编辑双链RNA，使腺嘌呤核苷（adenosine, A）经脱氨反应生成次黄嘌呤核苷（inosine, I）。ADAR1在非小细胞肺癌（non-small cell lung cancer, NSCLC）中的作用机制尚未明确。本研究拟分析ADAR1在NSCLC中的预后意义及其对癌细胞增殖迁移行为的影响。

**方法:**

基于癌症基因组图谱（The Cancer Genome Atlas, TCGA）和cBioPortal数据库，分析ADAR1高表达与肺癌临床病理特征及预后的关系；运用蛋白质免疫印迹（Western blot, WB）、细胞增殖检测、Transwell侵袭和迁移实验以及裸鼠皮下移植瘤模型，探索敲低ADAR1对肺癌细胞表型的影响及其潜在分子机制，进一步通过ADAR1 p150过表达模型验证该机制。

**结果:**

ADAR1在肺腺癌（lung adenocarcinoma, LUAD）和肺鳞癌（lung squamous cell carcinoma, LUSC）组织中的表达显著高于癌旁组织（LUAD: *P*=3.70×10^-15^, LUSC: *P*=0.016），其高表达与患者不良预后（LUAD: *P*=2.03×10^-2^, LUSC: *P*=2.81×10^-2^）及疾病远处转移密切关联（*P*=0.003）。基因富集分析（Gene Set Enrichment Analysis, GSEA）提示ADAR1高表达与丝裂原活化蛋白激酶/细胞外信号调节激酶（mitogen-activated protein kinase/extracellular signal-regulated kinase, MAPK/ERK）信号通路激活、基质金属蛋白酶-9（matrix metalloproteinase-9, MMP-9）表达及细胞黏附有关。在10株肺癌细胞系中，ADAR1与MMP-9蛋白水平呈显著正相关（*P*=6.45×10^-34^），且H1581细胞的ADAR1表达量最高。敲低H1581细胞中的*ADAR1*后，细胞形态趋于圆形、伪足减少，细胞增殖、侵袭、迁移及体内成瘤能力均减弱；同时，ERK磷酸化水平下降，细胞性FBJ骨肉瘤病毒癌基因同源物（cellular Finkel-Biskis-Jinkins murine osteosarcoma viral oncogene homolog, c-FOS）、MMP-9、N-cadherin、Vimentin表达减少。而在PC9细胞中过表达ADAR1 p150亚型后，ERK磷酸化水平升高，c-FOS和MMP-9表达增加。

**结论:**

ADAR1高表达与NSCLC患者不良预后及疾病远处转移密切关联。ADAR1可能经ERK/c-FOS/MMP-9轴调节肺癌细胞增殖、侵袭、迁移及体内成瘤能力。

## Abstract (en)

**Background and objective:**

Double-stranded RNA-specific adenosine deaminase 1 (ADAR1) binds to double-stranded RNA and catalyzes the deamination of adenosine (A) to inosine (I). The functional mechanism of ADAR1 in non-small cell lung cancer (NSCLC) remains incompletely understood. This study aimed to investigate the prognostic significance of ADAR1 in NSCLC and to elucidate its potential role in regulating tumor cell proliferation and migration.

**Methods:**

Data from The Cancer Genome Atlas (TCGA) and cBioPortal were analyzed to assess the correlation between high ADAR1 expression and clinicopathological features as well as prognosis in lung cancer. We performed Western blot (WB), cell proliferation assays, Transwell invasion/migration assays, and nude mouse xenograft modeling to examine the phenotypic changes and molecular mechanisms induced by ADAR1 knockdown. Furthermore, the ADAR1 p150 overexpression model was utilized to validate the proposed mechanism.

**Results:**

ADAR1 expression was significantly elevated in lung adenocarcinoma (LUAD) and lung squamous cell carcinoma (LUSC) tissues compared with adjacent non-tumor tissues (LUAD: *P*=3.70×10^-15^, LUSC: *P*=0.016). High ADAR1 expression was associated with poor prognosis (LUAD: *P*=2.03×10^-2^, LUSC: *P*=2.81×10^-2^) and distant metastasis (*P*=0.003). Gene Set Enrichment Analysis (GSEA) indicated that elevated ADAR1 was associated with mitogen-activated protein kinase/extracellular signal-regulated kinase (MAPK/ERK) pathway activation, matrix metalloproteinase-9 (MMP-9) expression, and cell adhesion. ADAR1 and MMP-9 levels showed a strongly positive correlation (*P*=6.45×10^-34^) in 10 lung cancer cell lines, highest in H1581. Knockdown of *ADAR1* in H1581 cells induced a rounded cellular morphology with reduced pseudopodia. Concomitantly, it suppressed cell proliferation, invasion, migration, and *in vivo* tumorigenesis. It also suppressed ERK phosphorylation and downregulated cellular Finkel-Biskis-Jinkins murine osteosarcoma viral oncogene homolog (c-FOS), MMP-9, N-cadherin, and Vimentin. Conversely, ADAR1 p150 overexpression in PC9 cells enhanced ERK phosphorylation and increased c-FOS and MMP-9 expression.

**Conclusion:**

High ADAR1 expression is closely associated with poor prognosis and distant metastasis in NSCLC patients. Mechanistically, ADAR1 may promote proliferation, invasion, migration, and tumorigenesis in lung cancer cells via the ERK/c-FOS/MMP-9 axis.

恶性肿瘤是人类的主要死因之一^[1]^，而肺癌是中国发病率（75.13/10万）和死亡率（51.94/10万）最高的癌种^[2]^。近年来抗肿瘤药物的研发和治疗组合策略的探索不断进行，肺癌的治疗手段迭代更新，显著改善了肺癌患者的预后，但由于肿瘤细胞的高度异质性、基因组不稳定性、表型可塑性等衍生了很多治疗难题，亟需人们加深对肿瘤致病分子机制的认识，探索多模式、个性化精准治疗策略^[3,4]^。

双链RNA特异性腺苷脱氨酶1（double-stranded RNA-specific adenosine deaminase 1, ADAR1）^[5]^是核苷脱氨酶家族的一员，通过结合和编辑双链RNA（double-stranded RNA, dsRNA），使腺嘌呤核苷（adenosine, A）经脱氨反应生成次黄嘌呤核苷（inosine, I）。ADAR1蛋白主要分为p110和p150两个亚型，p110亚型主要存在于细胞核中，维持细胞内天然存在的dsRNA和感受器的平衡状态；当细胞内的dsRNA失衡而出现堆积时，p150则发挥抑制dsRNA激活细胞固有免疫的主要功能^[6]^。ADAR1主要通过以下机制来影响肿瘤的发生和进展：一方面，ADAR1能抑制dsRNA激活细胞内的模式识别受体（pattern recognition receptors, PRRs），从而抑制细胞内固有免疫通路；另一方面，ADAR1对dsRNA的编辑会引起RNA突变，影响RNA的稳定性和功能，导致蛋白的表达量和功能发生变化^[7]^。

ADAR1在多种类型的肿瘤中存在上调现象，如前列腺癌、乳腺癌、肝细胞癌、非小细胞肺癌（non-small cell lung cancer, NSCLC）等^[8]^。已有研究表明，在不同的癌种中，ADAR1的上调会促进肿瘤细胞的复制和增殖^[9]^，抑制固有免疫通路^[10]^，促进肿瘤细胞逃避免疫清除^[11]^，影响免疫微环境^[12,13]^，促进肿瘤组织浸润和转移^[14]^，促进血管生成^[15]^，抑制细胞衰老^[16]^，抑制细胞坏死和凋亡，促进化疗耐药^[17]^和免疫治疗耐药^[18]^等。

本研究旨在探索ADAR1在NSCLC患者中的表达模式及其临床意义，基于公共数据库的生物信息学分析，系统挖掘ADAR1在NSCLC发生发展中的分子特征，并通过体外和体内实验对关键结果进行初步验证，以期为ADAR1作为NSCLC新型预后生物标志物和潜在治疗靶点提供理论依据。

## 1 材料与方法

### 1.1 细胞、试剂

肺癌细胞系NCI-H1581、NCI-H1838、NCI-H1563、NCI-H1650、PC9、HCC827、NCI-H1975、A549、NCI-H2347、NCI-H441和293T细胞由广东省肺癌研究所提供，购自美国菌种保藏中心（American Type Culture Collection, ATCC），并经过短串联重复序列（short tandem repeat, STR）鉴定，处于支原体阴性状态。试剂和材料包括：胎牛血清（10091148, Sigma-Aldrich）、细胞培养基（C22400500BT, Thermo Fisher）、磷酸盐缓冲液（41403ES76, Yeasen）、Trypsin胰酶（25200072, Thermo Fisher）、细胞裂解液（P0013B, Beyotime）、蛋白酶抑制剂（P0100, Solarbio）、磷酸酶抑制剂（4906837001, Roche）、蛋白质定量试剂盒（PC0020, Solarbio）、5×蛋白上样缓冲液（ZJ101, EpiZyme）、蛋白预染双色marker（WJ103, EpiZyme）、电泳缓冲液（G2081-15, Servicebio）、蛋白印迹转膜干粉（WB52002, NCM）、PVDF膜（Merck Millipore, IPVH00010）、化学发光液（P10300, NCM）、细胞计数试剂盒8（cell counting kit-8, CCK-8）（C6005, NCM）、抗体稀释液（WB100D, NCM）、杂交膜清洗液（WB20500, NCM）、无内毒素质粒中提试剂盒（P1155-03, Magen）、Polybrene（C0351-1ml, Beyotime）、Puromycin（ST551-50 mg, Beyotime）、基质胶（354234, BioCoat）、结晶紫染色液（C0121-500ml, Beyotime）、ADAR1抗体（82184S, CST）、甘油醛-3-磷酸脱氢酶（glyceraldehyde-3-phosphate dehydrogenase, GAPDH）抗体（PTM-6620, PTM BIO）、Vinculin抗体（13901S, CST）、基质金属蛋白酶-9（matrix metalloproteinase-9, MMP-9）抗体（13667T, CST）、细胞外信号调节激酶（extracellular signal-regulated kinase, ERK）抗体（4695S, CST）、磷酸化ERK（phospho-ERK, p-ERK）（Thr202/Tyr204）抗体（4376S, CST）、细胞性FBJ骨肉瘤病毒癌基因同源物（cellular Finkel-Biskis-Jinkins murine osteosarcoma viral oncogene homolog, c-FOS）[属于即刻早期基因（immediate early gene, IEG）]抗体（2250S, CST）、神经钙黏蛋白（neural cadherin, N-cadherin）抗体（13116S, CST）、Vimentin抗体（5741S, CST）。

### 1.2 cBioPortal肺癌数据下载和临床分期分析

从cBioPortal下载NSCLC的全外显子测序数据（whole-exome sequencing, WES）和临床数据，根据ADAR1的WES结果将队列分为*ADAR1*扩增组（低水平扩增、高水平扩增）和*ADAR1*未扩增组（二倍体、缺失），并使用卡方检验对组间远处转移和临床分期的构成比进行统计分析。

### 1.3 癌症基因组图谱（The Cancer Genome Atlas, TCGA）转录组数据分析

采用PanCanSurvPlot在线工具对肺腺癌（lung adenocarcinoma, LUAD）及肺鳞癌（lung squamous cell carcinoma, LUSC）患者进行生存分析。下载TCGA肺癌转录组数据，使用R（v4.1.1）完成以下分析：通过*Wilcoxon*检验比较ADAR1在肿瘤与正常组织中的表达差异；将513例LUAD样本按Xtile截断值划分为ADAR1高表达组（*n*=477）与低表达组（*n*=36），利用DESeq2包进行差异表达分析及可视化，clusterProfiler包对差异基因进行基因集富集分析（Gene Set Enrichment Analysis, GSEA），并对STRING数据库中的基因集（如“Positive regulation of MAPK cascade”）开展单通路富集分析。需说明的是，生存分析由PanCanSurvPlot工具基于其内置标准自动完成，与表达分析（R语言）的数据处理流程相互独立，数据处理及样本筛选差异导致二者的病例数不同，该情况不影响各分析内部的有效性与结论的可靠性。

### 1.4 免疫蛋白印迹（Western blot, WB）

取生长状态良好的细胞，经超声裂解后离心收集上清液，并测定蛋白浓度。取30 μg总蛋白与上样缓冲液混合，沸水浴变性后电泳分离蛋白样品（浓缩胶90 V，分离胶120 V），随后在含20%甲醇的转膜液中以300 mA恒定电流转膜120 min（时间依目的蛋白分子量酌情调整）。用5%脱脂牛奶封闭2 h后，依次孵育一抗和二抗，最后采用化学发光法显影。

### 1.5 慢病毒包装与稳转株构建

靶向ADAR1的shRNA（sh-ADAR1#1/#2/#3）及其空载对照（shRNA-EV）、ADAR1 p150过表达质粒（h-ADAR pCDH-GFP+Puro-3×FLAG）及其空载对照（pCDH-GFP+Puro-3×FLAG）均由湖南丰晖生物公司合成；psPAX2与pMD2.G包装质粒购自Addgene公司。慢病毒包装使用293T细胞进行：转染前一天将细胞铺板于6孔板中，待细胞密度达70%-80%时，使用Lipo3000转染试剂将相应质粒系统共转染入细胞，72 h后收集含病毒颗粒的上清液，用于感染靶细胞。感染1周后，使用嘌呤霉素对细胞进行筛选，直至细胞稳定增殖且荧光比例接近100%，随后提取总蛋白并通过WB验证蛋白表达效率。

### 1.6 绘制CCK-8增殖曲线

取生长状态良好的H1581 sh-ADAR1细胞株（包括对照组shRNA#EV，处理组sh-ADAR1#1与sh-ADAR1#2），经消化计数后，调整细胞密度至1.5×10^4^个/mL，以每孔100 µL接种于96孔板，每组设3个复孔。分别于培养第0、1、2、3天，更换培养基为含10% CCK-8与10%胎牛血清的完全培养基，37 °C孵育1-4 h后，使用酶标仪检测450 nm波长处吸光度。数据采用GraphPadPrism软件进行双因素方差分析，并绘制细胞增殖曲线。

### 1.7 Transwell迁移和侵袭实验

实验前一天使用基质胶包被8 µm Transwell小室。取生长状态良好的细胞，经消化计数后，用无血清培养基调整细胞密度至8×10⁵ 个/mL，向下室（24孔板）中加入800 µL含10%胎牛血清的完全培养基，上室（Transwell小室）中加入100 µL细胞悬液，培养48 h后取出Transwell小室，经4%多聚甲醛固定及结晶紫染色，于显微镜下观察并拍照记录。Transwell小室迁移实验使用未包被基质胶的8 µm Transwell小室，其余步骤同侵袭实验。

### 1.8 裸鼠皮下成瘤实验

取生长状态良好的细胞，经消化计数后用磷酸盐缓冲液调整细胞密度至2×10⁷个/mL。选用5周龄雄性BALB/c裸小鼠（由广东省医学实验动物中心提供），于每只裸鼠后腿皮下注射200 μL对照组或处理组的细胞悬液（含2×10⁶个细胞），以模拟体内肿瘤生长过程。定期观察小鼠成瘤情况，接种2周后以过量阿佛丁麻醉实施安乐死，取出皮下肿瘤，测量瘤体体积（体积公式：V=½×a×b²，其中a为长轴，b为短轴），瘤体经4%多聚甲醛固定后石蜡包埋备用。本实验严格遵守国家实验动物管理与使用规范，并获广东省医学实验动物中心动物伦理委员会批准（伦理批号：KY2025-624-02），所有操作均遵循美国国立卫生研究院（National Institutes of Health, NIH）《实验动物护理和使用指南》标准。

### 1.9 统计方法

所有图表均使用GraphPad Prism（version 9.5.0）或R语言（version 4.3.1）进行绘制。所有实验均独立重复3次。定量数据以均数±标准差（Mean±SD）表示，两组间比较采用*Student’s t*检验，多组间比较采用单因素方差分析，mRNA或蛋白表达水平之间的相关性采用*Pearson*相关性分析进行评估。*P*<0.05认为差异具有统计学意义。

## 2 结果

### 2.1 ADAR1在NSCLC组织中的表达差异

TCGA数据显示（图1A），ADAR1在LUAD和LUSC肿瘤组织中的表达均显著高于正常组织（LUAD: *P*=3.7×10^-15^, LUSC: *P*=0.016）。这一上调趋势在The Human Protein Atlas数据库的免疫组化结果中得到进一步验证（图1B）。使用PanCanSurvPlot在线分析工具对ADAR1高表达组和低表达组进行生存分析（图1C），在LUAD患者中，ADAR1高表达患者的无病间期（disease-free interval, DFI）显著缩短[风险比（hazard ratio, HR）=2.74，*P*=2.03×10^-2^]；在LUSC患者中，ADAR1高表达患者的总生存期（overall survival, OS）显著缩短（HR=1.40, *P*=2.81×10^-2^）。以上数据表明，ADAR1高表达与NSCLC患者的不良预后有关，ADAR1在NSCLC中可能发挥癌基因作用。

**图1:**
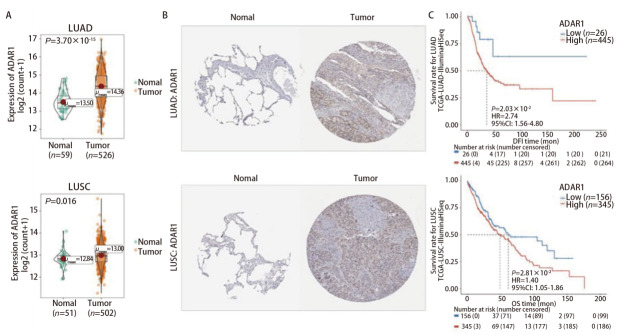
TCGA数据库中ADAR1的表达差异和预后差异。A：肿瘤和正常组织的ADAR1表达箱线图（数据来源：TCGA）；B：肿瘤和正常组织的ADAR1免疫组化图（图片来源：The Human Protein Atlas）；C：ADAR1高、低表达组患者的总体生存期与无病间期的Kaplan-Meier生存曲线（数据来源：TCGA；生存分析工具：PanCanSurvPlot）。

### 2.2 *ADAR1*扩增患者的临床分期特征

基于cBioPortal数据库的NSCLC WES数据，本研究将患者分为*ADAR1*未扩增型（*ADAR1* non-amp）和*ADAR1*扩增型（*ADAR1* amp），比较两组远处转移发生比例（图2A、2B）及晚期（III-IV期）诊断比例（图2C、2D）的差异。结果显示，在多个LUAD和LUSC独立队列中，*ADAR1*扩增型患者的远处转移发生率普遍高于未扩增型患者，其中Pan-lung cancer（TCGA, Nat Genet 2016）队列的差异具有统计学意义（*P*=0.003），其他队列虽未达显著水平，但整体趋势一致；同时，*ADAR1*扩增型患者在多个队列中也表现出更高的晚期（III-IV期）诊断比例，该趋势在Pan-lung cancer（TCGA, Nat Genet 2016）与LUSC（TCGA, PanCancer Atlas）两个队列中均具有统计学差异（*P*=0.018, *P*=0.023），其他队列虽未达显著水平，但整体趋势一致，表明*ADAR1*扩增与NSCLC的疾病进展及晚期临床特征密切关联。

**图2:**
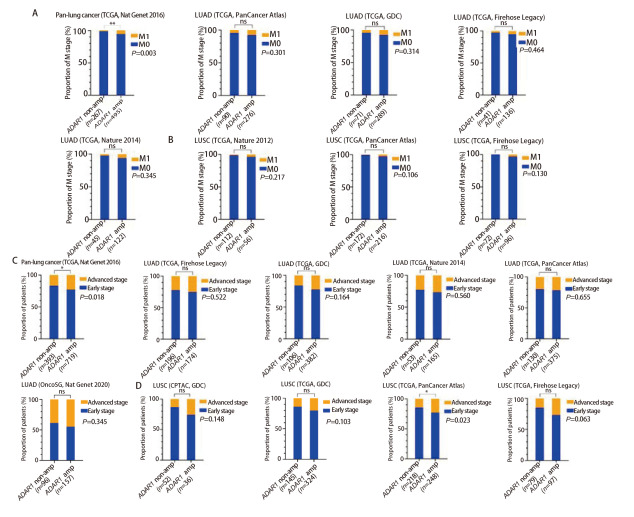
cBioportal数据库中ADAR1扩增与临床分期及转移的关系。A、B：肺腺癌和肺鳞癌队列中ADAR1未扩增组和ADAR1扩增组的M分期构成比图；C、D：肺腺癌和肺鳞癌队列中ADAR1未扩增组和ADAR1扩增组的临床分期（早期I-II期）与（晚期III-IV期）构成比图。

### 2.3 TCGA LUAD转录组数据的差异分析和富集分析

下载TCGA LUAD转录组数据，提取513例肿瘤组织的RNAseq数据，将样本分为ADAR1高表达组和ADAR1低表达组，对两组间的差异基因进行基因集富集分析（图3A），结果显示：ADAR1高表达组中显著富集的上调通路包括神经活性配体-受体相互作用、角质化包膜形成及cAMP信号通路等；而下调通路则主要涉及朊病毒疾病、化学致癌-活性氧及氧化磷酸化等。为探究ADAR1在肿瘤转移与侵袭中的作用，我们进一步分析了相关通路。MAPK通路关键激活分子的基因热图显示，其mRNA水平在ADAR1高表达样本中普遍上调（图3B）。基因集富集分析进一步证实，ADAR1高表达组中MAPK通路显著激活[标准化富集分数（normalized enrichment score, NES）=1.43，*P*=0.02]，且细胞黏附分子表达也明显上调（NES=2.80, *P*<0.001）（图3C、3D）。此外，相关性分析（图3E）结果显示，在mRNA层面，LUAD样本的MMP-9表达与*ADAR1*表达呈显著正相关（*r*=0.28, *P*=1.40×10^-10^）。

**图3:**
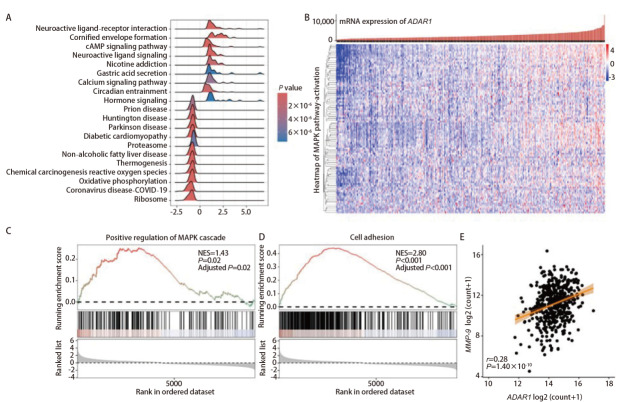
基于ADAR1分层的TCGA肺腺癌差异表达与富集分析。A：ADAR1高表达组和低表达组的差异基因的基因集富集分析，按照NES进行排序，展示前20个GSEA通路；B：样本按照ADAR1 mRNA水平的升序排序，显示MAPK通路激活分子相关差异表达基因热图，低水平表达的基因用蓝色表示，高水平表达的基因用红色表示；C、D：基因集“Positive regulation of MAPK cascade”与“Cell adhesion”的GSEA富集曲线图；E：TCGA肺腺癌转录组数据中ADAR1和MMP-9 mRNA表达水平的相关性分析。

### 2.4 ADAR1的基础表达和敲低细胞模型

选取10株LUAD细胞系（H1581、H1838、H1563、H1650、PC9、H827、H1975、A549、H2347、H441）进行培养，通过WB检测ADAR1和MMP-9的蛋白表达水平（图4A、4B）。结果显示，ADAR1主要包括两个亚型p150、p110，且H1581细胞中ADAR1表达量最高。相关性分析进一步表明（图4C），ADAR1与MMP-9蛋白表达呈显著正相关（*P*=6.45×10^-34^）。基于上述结果，选用ADAR1高表达的H1581细胞构建*ADAR1*稳定敲低模型。WB验证显示（图4D、4E），与对照组（shRNA#EV）相比，sh-ADAR1#1与sh-ADAR1#2组的ADAR1蛋白水平分别下降67.5%（*P*=0.0002）与61.3%（*P*=0.0102），表明敲低模型构建成功。形态学观察发现，*ADAR1*敲低后细胞由近似纺锤形转变为鹅卵石形，伪足减少，失去典型间质细胞特征（图4F）。

**图4:**
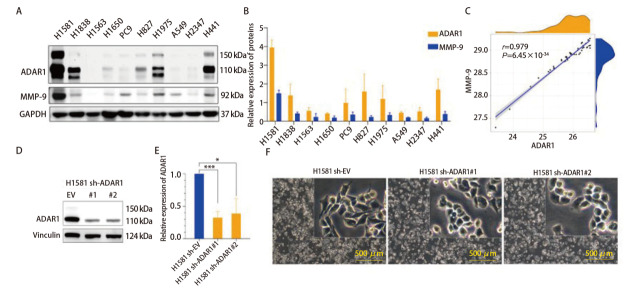
构建shRNA稳定敲低ADAR1的H1581细胞模型。A：WB验证肺腺癌细胞系ADAR1和MMP9蛋白的基础表达量；B、C：肺腺癌细胞系ADAR1和MMP9蛋白的基础表达量的灰度值分析和相关性分析；D、E：WB验证H1581 sh-ADAR1细胞模型的蛋白敲低水平；F：H1581 sh-ADAR1细胞模型的细胞形态变化。

### 2.5 *ADAR1*敲低抑制肺癌细胞系的增殖、皮下成瘤、侵袭和迁移能力

为探究ADAR1对肺癌细胞恶性表型的影响，构建H1581-shADAR1稳定转染细胞系并进行功能验证。CCK-8增殖实验显示（图5A），与对照组（H1581 shRNA#EV）相比，两个敲低组（H1581 sh-ADAR1#1与#2）的吸光度值均显著降低（*P*<0.0001），表明*ADAR1*敲低后细胞增殖速率明显减慢。进一步通过皮下移植瘤实验评估ADAR1对体内成瘤能力的影响（图5B），接种细胞后，与对照组相比，sh-ADAR1#1与sh-ADAR1#2组的皮下成瘤速度明显减慢，2周后对小鼠实施安乐死并取出瘤体，使用卡尺测量法得到小鼠的终点瘤体积，结果显示，sh-ADAR1#1与sh-ADAR1#2组的相对瘤体体积分别下降至0.098（*P*=0.0015）与0.057（*P*<0.0001），表明*ADAR1*敲低显著抑制了肿瘤生长（*n*=4）。Transwell实验结果显示（图5C、5D），*ADAR1*敲低后，细胞的侵袭与迁移能力均显著减弱（*P*<0.0001）。以上结果表明，ADAR1在维持肺癌细胞增殖、成瘤、侵袭及迁移能力中发挥关键作用。

**图5:**
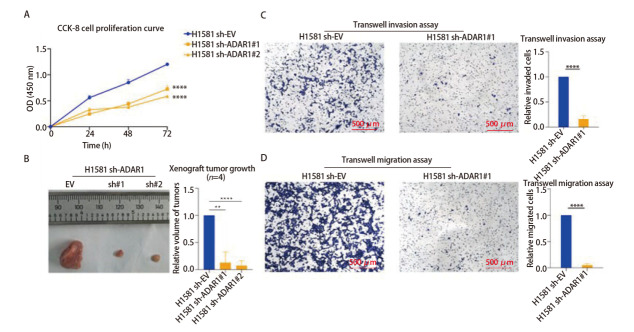
H1581细胞稳定敲低ADAR1后的表型实验。A：H1581 sh-ADAR1细胞模型3 d的CCK-8增殖曲线；B：H1581 sh-ADAR1细胞模型的裸鼠皮下成瘤实验；C：H1581 sh-ADAR1细胞模型的Transwell侵袭实验；D：H1581 sh-ADAR1细胞模型的Transwell迁移实验。

### 2.6 ADAR1通过ERK/c-FOS通路影响MMP-9蛋白表达

为阐明ADAR1在ERK通路及肿瘤转移中的作用，通过WB检测了*ADAR1*敲低后H1581细胞中相关蛋白的表达变化（图6A）。结果显示，与阴性对照组（shRNA#EV）相比，两个*ADAR1*敲低组（sh-ADAR1#1和#2）中MMP-9、c-FOS、N-cadherin及Vimentin的蛋白表达水平均显著下降，ERK磷酸化水平也明显降低（均*P*<0.05）。为进一步验证ADAR1的功能，在PC9细胞中构建了ADAR1 p150亚型的过表达稳转株（图6B）。与空载对照组（PC9 EV）相比，PC9 ADAR1 p150过表达组中MMP-9、c-FOS的表达及ERK磷酸化水平均显著上调（均*P*<0.05）。上述结果共同表明，ADAR1总蛋白的敲低可抑制ERK/c-FOS通路的活化并下调MMP-9等转移相关蛋白的表达，而ADAR1 p150过表达会导致细胞内ERK/c-FOS通路激活，MMP-9蛋白表达上调。

**图6:**
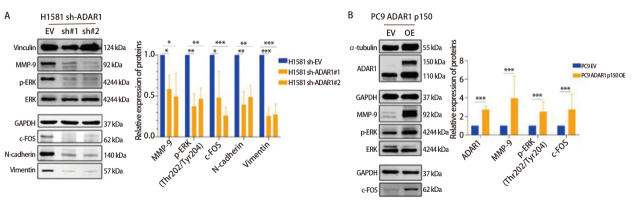
WB验证ADAR1敲低后细胞ERK通路和转移相关分子的表达变化。A：WB验证H1581 sh-ADAR1细胞模型的MMP-9、p-ERK、ERK、c-FOS、N-cadherin、Vimentin蛋白表达量的变化；B：PC9 ADAR1 p150 OE细胞模型的MMP-9、p-ERK、ERK、c-FOS蛋白表达量的变化。

## 3 讨论

ADAR1已被广泛报道在多种恶性肿瘤中参与肿瘤转移和疾病进展的调控。在前列腺癌中，ADAR1介导的RNA编辑能够影响多个关键分子的功能，包括促进抗酶抑制剂1（antizyme inhibitor 1, AZIN1）向细胞核内转移^[19]^、调节DNA聚合酶α亚基B（DNA polymerase alpha subunit B, POLA2）的蛋白功能及其作用网络^[20]^、调节异黏附素（metadherin, MTDH）^[9]^的蛋白表达水平，这些分子事件进一步激活包括磷脂酰肌醇-3-激酶/蛋白激酶B（phosphoinositide 3-kinase/protein kinase B, PI3K/AKT）、核因子κB（nuclear factor kappa-B, NF-κB）、MAPK和Wnt/β-catenin在内的多条信号通路，从而增强肿瘤细胞的存活与侵袭能力。在胃癌中，ADAR1不仅通过调控AZIN1诱导上皮-间质转化（epithelial-mesenchymal transition, EMT）过程^[21]^，还激活哺乳动物雷帕霉素靶蛋白（mammalian target of rapamycin, mTOR）信号通路以促进肿瘤恶性进展^[22]^。在肝细胞癌中，ADAR1通过上调整合素亚基α2（integrin subunit alpha 2, ITGA2）的表达增强肿瘤细胞对细胞外基质的黏附能力^[23]^，此外，其介导的miR-3144-3p编辑可间接调控Musashi RNA结合蛋白2（Musashi RNA-binding protein 2, MSI2）和溶质载体家族38成员4（solute carrier family 38 member 4, SLC38A4）的表达，进而影响细胞EMT进程^[24]^。在NSCLC中ADAR1通过调控辐射敏感蛋白18（radiation sensitive 18, *Rad18*）基因表达，增强肿瘤细胞的DNA损伤修复能力，从而赋予其放疗抵抗性^[25]^，临床分析显示，ADAR1的高表达与淋巴结转移显著相关，并伴随着淋巴结转移灶内CD4^+ ^T细胞和M1巨噬细胞数量下降，提示其参与免疫微环境的重塑^[26]^；此外，ADAR1通过对细胞色素P450家族1亚族A成员1（cytochrome P450 family 1 subfamily A member 1, CYP1A1）转录本的编辑通过激活PI3K/AKT通路增强肿瘤细胞的抗氧化应激能力并促进其侵袭能力^[27]^；ADAR1还能通过稳定黏着斑激酶（focal adhesion kinase, FAK）转录本促进肿瘤细胞骨架重组，进而增强肿瘤的转移潜能^[28]^。

基于上述研究背景，本研究重点探讨了ADAR1高表达与NSCLC远处转移及不良预后的关联性。首先，基于公共数据库的分析结果显示，在LUAD和LUSC组织中ADAR1的表达量显著高于正常组织，且ADAR1高表达与较短生存时间显著关联。进一步对cBioPortal中NSCLC队列的分析表明，*ADAR1*扩增型患者的远处转移发生率显著提高、临床晚期（III-IV期）的诊断比例也显著提高。此外，通过对TCGA LUAD表达谱数据的差异分析和GSEA发现，在mRNA层面*ADAR1*的上调与MAPK通路激活分子及细胞黏附分子的表达升高显著关联，且*ADAR1*表达与*MMP-9*呈正相关。

为验证上述发现，本研究分别通过体外及体内实验进行功能探究。首先，在10株LUAD细胞系中检测了ADAR1和MMP-9的蛋白表达水平，*Pearson*相关性分析显示两者表达呈显著正相关，其中H1581细胞系的ADAR1和MMP-9表达均为最高，故将其作为后续实验模型。在构建*ADAR1*稳定敲低的H1581细胞模型后，CCK-8增殖检测、Transwell侵袭和迁移实验及裸鼠皮下成瘤实验的结果表明：*ADAR1*的敲低会导致H1581的细胞形态变圆、细胞伪足减少，细胞的增殖、侵袭和迁移能力及体内成瘤能力减弱。在分子机制层面，WB结果显示，ADAR1敲低后ERK通路磷酸化水平降低，转录因子c-FOS、MMP-9和细胞黏附分子（N-cadherin、Vimentin）表达均下调。

基于TCGA LUAD转录组数据绘制的差异基因热图显示，在ADAR1高表达样本中，MAPK通路相关激活分子的mRNA水平普遍上调，涉及多个功能类别，包括MAPK通路成员（如MAP2K、MAP3K、MAPK）、成纤维细胞生长因子（fibroblast growth factor, FGF）家族成员（如FGF3、FGF8、FGF17、FGF22）、胰岛素样生长因子（insulin-like growth factor, IGF）家族成员（如IGF1、IGF2）、胰岛素生长因子1受体（insulin-like growth factor 1 receptor, IGF1R）以及血小板衍生生长因子受体β（platelet-derived growth factor receptor beta, PDGFR-β）、转化生长因子-β（transforming growth factor beta, TGF-β）和上皮细胞有丝分裂原（epithelial mitogen, EPGN）等。本课题组推测，ADAR1可能通过其dsRNA的结合和编辑功能，增强了这些mRNA的稳定性或翻译效率，从而促进相应蛋白的表达、进一步激活ERK通路并上调转录因子c-FOS，促进激活蛋白-1（activator protein-1, AP-1）直接结合MMP-9启动子区的AP-1位点，从而促进MMP-9的转录表达，最终增强细胞的增殖、迁移和侵袭能力。在功能验证实验中，H1581细胞的*ADAR1*敲低通过抑制ERK/c-FOS/MMP-9信号轴，显著削弱了细胞的增殖和侵袭能力；相反，在肺癌细胞系PC9中过表达ADAR1的全长亚型p150后，细胞的ERK磷酸化水平上调，c-FOS和MMP-9表达亦随之上升，这共同验证了ADAR1对ERK/c-FOS/MMP-9通路具有调控作用，并提示p150亚型在该机制中发挥了重要作用。

此外，细胞形态由近似纺锤体样变为鹅卵石样，细胞形态变圆、细胞伪足减少，提示细胞间质特性减弱与黏着斑形成受阻，伴随细胞转移相关黏附分子N-cadherin、Vimentin表达下调，进一步提示细胞间质特征减弱，这可能是ADAR1调控肿瘤细胞增殖与侵袭表型的另一重要机制。

综上所述，本研究从公共数据库挖掘、体外细胞实验和体内动物模型3个层面系统阐明了ADAR1在NSCLC恶性进展中的作用。研究结果表明，ADAR1通过激活ERK/C-FOS信号轴，上调MMP-9表达并促进细胞黏附分子表达，从而增强NSCLC细胞的增殖、成瘤、侵袭和迁移能力。本研究的创新性在于首次揭示了ADAR1与ERK/c-FOS/MMP-9轴之间的功能联系，为理解ADAR1促进肺癌进展的分子机制提供了新的视角，也为该基因作为预测NSCLC预后不良与转移风险的潜在生物标志物提供了实验依据。值得注意的是，ERK通路作为肿瘤研究中的重要信号通路，在多种癌症进展中发挥关键作用，且针对该通路的药物研发已取得一定进展。因此，本研究揭示的ADAR1与ERK通路之间的调控关系，或可为ADAR1高表达患者从ERK靶向治疗中获益提供理论参考。然而，本研究仍存在若干局限性：在临床层面，尚缺乏基于大规模临床队列的ADAR1表达差异与临床病理特征关联性分析；在实验研究层面，当前对细胞模型生物学行为的考察仍不系统，体内实验仅采用终点瘤体积评估肿瘤生长，未能通过动态监测完整呈现肿瘤演进过程，也尚未评估ADAR1对肿瘤全身性转移的影响；在分子机制层面，ADAR1调控ERK通路的关键靶点仍未明确，现有数据主要基于生物信息学角度提示ADAR1可能通过促进MAPK通路中多个激活分子的转录水平上调发挥作用。

针对上述局限，后续研究将着重从以下方面展开：首先，深入解析ADAR1调控ERK信号通路的具体分子机制，明确其关键作用靶点；其次，拓展细胞模型的表型研究范围和深度，增设处理因素梯度，以提供过更全面、计量依赖的实验证据；同时，通过尾静脉注射转移模型结合活体成像技术，系统评估ADAR1在小鼠全身转移中的作用，对小鼠成瘤进展及生理状态进行动态监测；最后，整合多中心临床样本，进一步验证ADAR1表达与临床病理特征及预后的相关性，为其临床转化提供更有力的证据支持。
